# Smear positive pulmonary tuberculosis and associated factors among homeless individuals in Dessie and Debre Birhan towns, Northeast Ethiopia

**DOI:** 10.1186/s12941-016-0165-x

**Published:** 2016-08-31

**Authors:** Tsedale Semunigus, Belay Tessema, Setegn Eshetie, Feleke Moges

**Affiliations:** 1 Amhara Regional Health Bureau, North Shewa Zonal Health Bureau, Debre Birhan, Ethiopia; 2 School of Biomedical and Laboratory Sciences, Department of Medical Microbiology, University of Gondar, P.O. Box: 196, Gonder, Ethiopia

**Keywords:** Homeless individuals, Tuberculosis, Associated factors, Northeast Ethiopia

## Abstract

**Background:**

Tuberculosis (TB) remains one of the globe’s deadliest communicable diseases. The homeless individuals are at high risk to acquire TB and multi-drug resistant TB (MDR-TB), because of their poor living conditions and risky behaviors. Tuberculosis and MDR-TB in the homeless individuals can pose a risk to entire communities. However, the magnitude of the problem is not known in Ethiopia. Therefore, the aim of this study was to determine the prevalence and associated factors of smear positive pulmonary TB (PTB) and MDR-TB among homeless individuals in Dessie and Debre Birhan towns, Northeast Ethiopia.

**Methods:**

A community based cross-sectional study design was conducted from September 2014 to June 2015. Using an active screening with cough of ≥2 weeks, 351 TB suspects homeless individuals were participated in this study. Data were collected by using pre-tested and structured questionnaire. Spot**-**morning**-**spot sputum sample was collected and examined for acid-fast bacilli (AFB) using fluorescence microscopy by Auramine O staining technique. All AFB positive sputum was further analyzed by GeneXpert for detection of *Mycobacterium tuberculosis* complex and rifampicin resistant gene. Univariate and multivariate logistic regressions were applied to identify factors associated with smear positive PTB and P value <0.05 was considered as statistically significant.

**Results:**

The prevalence of smear positive PTB was 2.6 % (95 % CI 1.3–5) among TB suspect homeless individuals. Extrapolation of this study finding implies that there were 505 smear positive PTB per 100,000 homeless individuals. All smear positive PTB sputum specimens were further analyzed by GeneXpert assay, the assay confirmed that all were positive for MTBC but none were resistant to RIF or MDR. Smoking cigarette regularly for greater than 5 years (AOR 10.1, 95 % CI 1.1, 97.7), body mass index lower than 18.5 (AOR 6.9, 95 % CI 1.12, 41.1) and HIV infection (AOR 6.8, 95 % CI 1.1, 40.1) were significantly associated with smear positive PTB.

**Conclusion:**

The prevalence of smear positive PTB among TB suspect homeless individuals was 2.6 %. Among smear positive PTB, prevalence of HIV co-infection was very high 5 (55.5 %). Smoking cigarette regularly for greater than 5 years, BMI lower than 18.5 and HIV infection were factors associated with smear positive PTB. Special emphasis is needed for homeless individuals to exert intensive effort to identify undetected TB cases to limit the circulation of the disease into the community.

## Background

Tuberculosis (TB) is an airborne chronic infectious disease mainly caused by *Mycobacterium tuberculosis* (MTB). The tubercle bacilli are obligate aerobes; grow most successfully in areas of the body with lots of blood and oxygen and commonest point of entry into the body is via the lungs, pulmonary TB (PTB), but may also affect any organ or tissue outside of the lungs, extra pulmonary TB (EPTB) [1]. Tuberculosis is mostly transmitted by inhalation of infected droplet nuclei, which are discharged in the air when a person with untreated PTB coughs, sneezes, spits and sings [2]. Globally PTB accounts for 85 % of all TB cases; among them smear positive PTB comprises 75–80 %. Smear positive PTB is the most infectious and most likely transmit from human to human and the infection prevention and control programs are air borne precautions. Therefore, the identification of TB suspects (cough for 2 weeks and more duration) and screening them by examination of sputum allows discovering those who are transmitting the disease and to start early treatment [3].

Poverty, malnutrition, over-crowded or unsanitary living conditions, low socioeconomic status, drug abuse, cigarette smoking, alcoholism, close contact with active TB cases, human immunodeficiency virus/acquired immune deficiency syndrome (HIV/AIDS) and increasing numbers of homeless people are the greatest risk factors for the acquisition of active TB [1]. Homelessness is a global problem; an estimated one hundred million to one billion people are homeless worldwide [4]. Homelessness is becoming a common feature of cities and fast growing towns of the poor countries in Africa, mainly due to a very high increasing rate of rural–urban migration and poverty [5]. Similarly, homeless people are also increasingly encountered in different area of Ethiopian cities [6]. Because of poor living conditions and as they indulge in risky behaviors, homeless people are exposed to many communicable diseases [7]. The death rate among this group of people is about 4 times higher than the general population [8]. Homeless people are included in the high-risk classification for developing TB disease by Centers for Disease Control and prevention (CDC) as they suffer disproportionately from a variety of health problems and emergency shelters remain volatile TB transmission sites [9].

In addition, being homelessness are creating favorable conditions for the development and transmission of MDR-TB because these groups are hard-to-reach groups, poor diagnostic and treatment services, more likely to incomplete and inadequate TB treatment and poor management of the disease including infection control [10, 11]. Many homeless TB patients often could not regard their health as a high priority and may prioritize substance needs such as food, shelter and providing for any addiction [12]. Globally, PTB in homeless individuals is especially problematic because it may be highly contagious and can present as advanced disease with poor outcomes, including mortality [13].

Generally 72 % of domestic TB outbreaks investigated by CDC in the year 2002–2010 involved homelessness in developed countries [14]. Therefore, appropriate health interventions should be done for homeless individuals to reduce the adverse outcomes of these communicable diseases [15]. Currently, Ethiopia is working towards interrupting transmission of TB, and preventing the emergence and spread of MDR-TB in the general population. In spite of these efforts, the problem remained a continuous challenge in the country [16]. Although, homelessness is one of the greatest risk factors for the acquisition of TB, and homelessness is a problem in Ethiopian cities, tuberculosis prevalence and associated factors among homeless individuals in Ethiopia has not been well reported. Therefore the aim of this study was to determine smear positive pulmonary tuberculosis and associated factors among homeless individuals in Dessie and Debre Birhan towns, Northeast Ethiopia.

## Methods

### Study area, study design and study participants

A community based cross-sectional study was conducted in Dessie and Debre Birhan towns from September 2014 to June 2015. Homeless individuals, who were aged ≥15 years, and had cough of 2 weeks and more duration were included in the study, whereas those who were unable to produce sputum were excluded from the study.

### Variables

#### Dependent variable

Smear positive PTB and MDR-TB.

#### Independent variable

Socio demographic factors: age, sex, marital status, religion, educational status. Behavioral factors: smoking, duration of smoking, alcohol drinking, duration of alcohol drinking, khat chewing, duration of khat chewing, drug using, duration of drug usage. Environmental factors: duration of being homeless, number of homeless individuals slept/live together in one restricted place, close contact with known TB patients, close contact with chronically cougher patients. Morbidity history and status: current TB suggestive symptoms, past TB history, past TB treatment starting, completion of TB treatment, body mass index (BMI), HIV infection.

### Sample size determination and sampling technique

A total of 351 individuals were enrolled in the study using active screening strategies to identify PTB suspects. Approximately 1780 homeless individuals were screened during the study period for symptoms suggestive of TB, such as cough of 2 weeks or more duration according to the National TB manual [17]. Out of the total screened, 351/1780 (19.7 %) homeless individuals were having cough of ≥2 weeks duration, were included into the study.

### Questionnaire

A structured and pre-tested questionnaire was completed by 4 trained data collectors (2 laboratory technologists and 2 nurses) by face-to-face interview. The questionnaire had four parts; socio demographic characteristics, behavioral characteristics, environmental factors and morbidity history and status of the study participants. A questionnaire was first developed in English and then translated into Amharic language for appropriateness and clarity so; the participants were interviewed with their mother languages and finally retranslated to English by another language expert to check its consistency.

### Sputum sample collection and florescence microscopy examination

About 3–10 ml of spot**-** morning**-**spot sputum samples were collected using coded and new, translucent, screw-capped specimen collection containers by laboratory technologist from the study participants. The sputum samples were placed in cold boxes immediately upon receipt and delivered to Dessie and Debre Birhan referral hospitals laboratory on the day of collection. Sputum-smear microscopy was performed using Primo Star iLED, light emitting diode (LED)—florescence microscopy (FM) by using Auramine O staining procedure as follows; a smear was prepared and dried, then heat-fixed. Stained the smear with filtered 0.1 % Auramine O solution and kept the staining reagent for 20 min and washed well. Decolorized with 0.5 % acid-alcohol and kept for 3 min and gently rinsed with water. Counterstained with 0.5 % potassium permanganate solution for 1 min, then gently rinsed with water and drained. Finally, the back of the slide cleaned, air-dried and the stained slides were observed under 20×, 40× magnifications of FM for AFB. The AFB was appeared bright yellow against dark background materials [18].

### GeneXpert examination

All AFB positive sputum samples were subjected to GeneXpert MTB/RIF system (Cepheid, USA) in Dessie and Debre Birhan referral hospitals laboratory. The system is a fully automated nested real-time polymerase chain reaction (PCR), which simultaneously detects *M. tuberculosis* complex (MTBC) and mutations in the ribonucleic acid polymerase Beta subunit gene (rpoB), which are responsible for the resistance to rifampin (RIF) [19–21].

### Rapid HIV test

To determine the HIV status of the study participants, pre-test counseling was provided by trained nurses. Then whole blood was collected by finger stick. The presence of HIV-1 and HIV-2 antibodies was determined by using rapid test kits, HIV (1  +  2) antibody Colloidal Gold (KHB, Shanghai Kehua Bio-engineering Co Ltd, China) as a screening test, followed by HIV 1/2 STAT-PAK® (Chembio Diagnostics, USA), when the KHB result was reactive. Where the result of STAT-PAK® was discordant with KHB, a third test, Unigold™ HIV (Trinity Biotech, Ireland), was also used as a tiebreaker to determine the test result following the manufacturers’ instruction. After testing, post test counseling was provided for all participants.

### Nutritional assessment

The participants’ body weight and height were measured by digital scale to the nearest 0.1 kg and 0.1 cm respectively. Body mass index is defined as the weight in kilogram by the individual divided by the square of the height in meter. It is used to determine the nutritional status of study participants into malnutrition (BMI  =  less than 18.5 kg/m^2^), normal (BMI  =  18.5–24.9 kg/m^2^) and overweight (BMI  =  25.0–29.9 kg/m^2^) as recommended by CDC [22].

### Quality assurance

A pre-test was done in Kombolcha town in 20 (5 %) homeless individuals who were similar with study participants prior to the data collection to check the clarity and consistency of the questionnaires and acceptability of laboratory procedure. Necessary correction was taken before the actual data collected. The data collectors, who can speak the local language (Amharic), were trained for 1 day on data collection procedures for this study to attain standardization and maximize interview reliability. The purpose of the study was informed to study participants for the quality of the data. In addition the study participants were instructed on how to produce an appropriate sputum specimen. Instruments and reagents were checked for reliability and reproducibility of the test before any test started. All new lots of reagents were tested with known positive and negative control. All positive microscopy slides and 10 % percent of negative slides were double checked by second experienced laboratory technologists for confirmation. The data collections, application of standard laboratory test procedures and test result were checked by senior laboratory technologist and principal investigator. Filled questionnaire and laboratory test result were collected after checking consistency and completeness. The overall data collection process was supervised by the principal investigator.

### Data processing and analysis

Following the data collection, data were checked, coded and entered using EPI-INFO version 3.5 and exported to SPSS version 20 for analysis. Both descriptive and analytical statistical procedures were utilized. Descriptive statistics like percentage, mean and standard deviation were used for presentation of data and prevalence of smear positive PTB and MDR-TB. All variables of the study were initially tested for association with smear positive PTB by using binary logistic regression model. Those variables which have a p-value less than 0.2 by univariate analysis were put in the multivariable analysis model to control the possible effect of confounders. Finally the variable which has independent association with smear positive PTB was identified on the basis of odd ratio (OR) with 95 % confidence interval (CI) and P value less than 0.05. The variable was entered into multivariate model using the forward stepwise (likelihood ratio) regression method. Model fitness was checked using Hosmer and Lemeshow goodness of a fit test (0.70).

## Results

### Socio-demographic characteristics of the study participants

A total of 351 individuals were enrolled, who had cough of 2 weeks or more duration. Out of the total study participants, 190 were from Dessie town that constituted 54.1 % of the total participants and 161 were from Debre Birhan town that comprised 45.9 % of the 351 participants. Majority of study participants were males, 324 (92.7 %), and 333 (95.9 %) were between 15 and 44 years of old. The mean age of the participants were 26.7 (SD ±7.96) years. About 163 (46.4 %) of the study participants were illiterate and most of the participants 308 (87.7 %) were single and 301 (85.7 %) of the participants followed Orthodox Christians religion (Table 1).

**Table 1:** Socio-demographic characteristics of homeless individuals with smear positive PTB prevalence, Dessie and Debre Birhan towns, Northeast Ethiopia, September 2014 to June 2015 (N = 351)

Variables	Smear positive PTB		Total N (%)
	Negative n (%)	Positive n (%)	
Age			
15–24	158 (46.2)	3 (33.3)	161 (45.9)
25–34	138 (40.3)	4 (44.4)	142 (40.5)
35–44	28 (8.2)	2 (22.3)	30 (8.5)
45–60	18 (5.3)	0 (0)	18 (5.1)
Sex			
Male	316 (92.4)	8 (88.9)	324 (92.3)
Female	26 (7.6)	1 (11.1)	27 (7.7)
Marital status			
Single	300 (87.7)	8 (88.9)	308 (87.8)
Married	11 (3.2)	1 (11.1)	12 (3.4)
Divorced	17 (4.9)	0 (0)	17 (4.8)
Widowed	14 (4.1)	0 (0)	14 (4.0)
Educational status			
Illiterate	158 (46.2)	5 (55.6)	163 (46.4)
Primary school	176 (51.5)	4 (44.4)	180 (51.3)
Secondary school	8 (2.3)	0 (0)	8 (2.3)
Religion			
Orthodox	293 (85.7)	8 (88.9)	301 (85.8)
Muslim	47 (13.7)	1 (11.1)	48 (13.7)
Protestant	2 (0.6)	0 (0)	2 (0.5)

### Prevalence of smear positive PTB and MDR-TB in homeless individuals

Out of the total study participants, smear positive PTB was detected in 9 of the participants (8 males and 1 female) by LED**-**FM. All smear positive PTB sputum specimens were further analyzed by GeneXpert assay, the assay confirmed that all were positive for MTBC but none were resistant to RIF or MDR. Therefore, the prevalence of smear positive PTB was 2.6 % (95 % CI 1.3, 5 %) among the study participants. The point prevalence of smear positive PTB was extrapolated to be 505/100,000 homeless individuals. All smear positive PTB cases were found in the age group (17–44 years) of the participants. Eight (88.9 %) smear positive PTB cases were found in the study participants who smoke cigarettes, drink alcohol and chew khat (flowering plant and leaves are chewed, contains cathinone, an amphetamine-like stimulant, which is said to cause excitement) during the study periods. Five (55.5 %) smear positive PTB cases were found in the study participants who were malnourished during the study periods. In addition, among the total smear positive PTB participants, 5 (55.5 %) were co-infected with HIV infection.

### Behavioral characteristics of the study participants

**Fig. 1:**
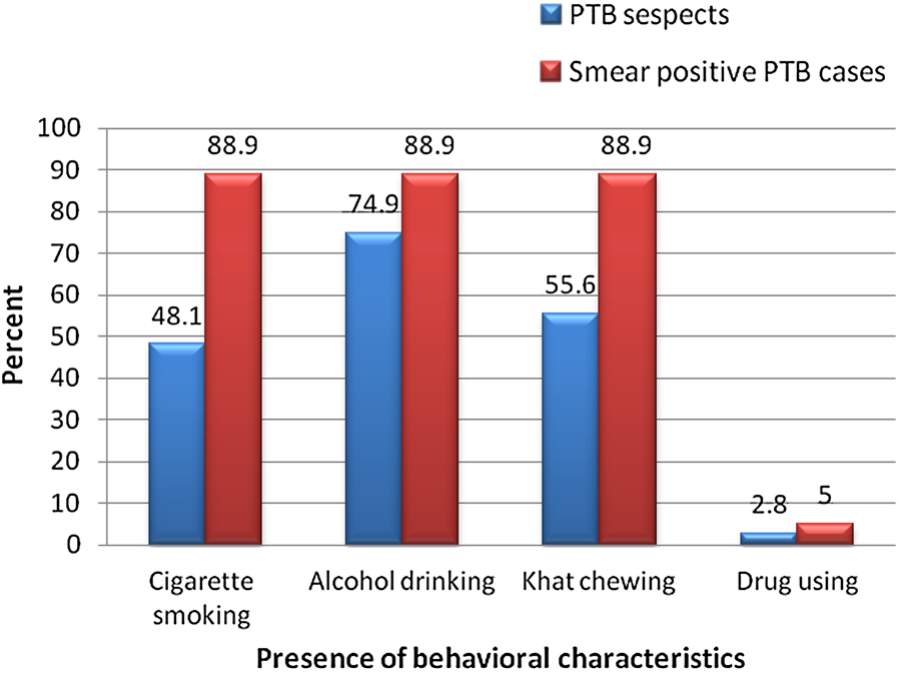
Behavioral characteristics of homeless individuals, Dessie and Debre Birhan towns, Northeast Ethiopia, September 2014 to June 2015 (N = 351)

Out of the total study participants 169 (48.1 %) had smoking cigarette during the study periods, of these, 67 (39.6 %) regularly used cigarette for greater than 5 years. The mean smoking periods of the participants was 64.7 (SD ±43.6) months. Besides, 263 (74.9 %) participants had experience with drinking alcohol, from these individuals, 164 (62.4 %) were regular alcohol drinkers for greater than 5 years. The mean alcohol drinking periods of the participants was 81.9 (SD ±56.9) months. More than half of the respondents, 195 (55.6 %) were also khat chewers, during the study periods. The mean khat chewing periods of the participants was 64.1 (SD ±46.2) months. About 10 (2.8 %) of the participants were using drugs during the study periods (Fig. 1).

### Living conditions of the homeless study participants

The participants mean duration of being homelessness was 65.9 (SD ±49.16) months and about 148 (42.2 %) of the participants were homelessness for greater than 5 years. The average number of homeless individuals slept/live together in one restricted homeless shelter were 5 (SD ±2.99) and almost half 163 (46.4 %) of the participants were sleeping/living together in one restricted homeless shelter by being more than 5 persons. Among the study participants 17 (4.8 %) and 114 (32.5 %) had close contact with known TB patients and chronically cougher patients respectively (Table 2).

**Table 2:** Environmental factors of homeless individuals with smear positive PTB prevalence, Dessie and Debre Birhan towns, Northeast Ethiopia, September 2014 to June 2015 (N = 351)

Variables	Smear positive PTB		Total N (%)
	Negative n (%)	Positive n (%)	
Duration of being homelessness			
>5 years	141 (41.2)	7 (77.8)	148 (42.2)
≤5 years	201 (58.8)	2 (22.2)	203 (57.8)
Average number of homeless			
>5 persons	157 (45.9)	6 (66.7)	163 (46.4)
≤5 persons	185 (54.1)	3 (33.3)	188 (53.6)
Close contact with known TB patients			
Yes	15 (4.4)	2 (22.2)	17 (4.8)
No	327 (95.6)	7 (77.8)	334 (95.2)
Close contact with chronically cougher			
Yes	108 (31.6)	6 (66.7)	114 (32.5)
No	234 (68.4)	3 (33.3)	237 (67.5)

### Morbidity history and status of the study participants

Out of the total study participants 10 (2.8 %) participants had a past history of TB disease, all of these were diagnosed during being homelessness and all were starting anti-TB treatment but more than half of them 6 (60 %) were defaulted anti-TB treatment. Out of the total study participants 25 (7.1 %) participants were malnourished, BMI less than 18.5. Out of the total study participants 22 (6.3 %) participants were HIV infected (Table 3).

**Table 3:** Morbidity history and status of homeless individuals with smear positive PTB prevalence, Dessie and Debre Birhan towns, Northeast Ethiopia, September 2014 to June 2015 (N = 351)

Variables	Smear positive PTB		Total N (%)
	Negative n (%)	Positive n (%)	
Past history of PTB			
Yes	9 (2.6)	1 (11.1)	10 (2.8)
No	333 (97.4)	9 (88.9)	341 (97.2)
Past anti-TB treatment			
Defaulted	5 (55.6)	1 (100)	6 (60)
Completed	4 (44.4)	0 (0)	4 (40)
BMI (kg/m^2^)			
<18.5	20 (5.8)	5 (55.6)	25 (7.1)
≥18.5	322 (94.2)	4 (44.4)	326 (92.9)
HIV antibody test			
Reactive	17 (5)	5 (55.6)	22 (6.3)
Non-reactive	325 (95)	4 (44.4)	329 (93.7)

### Factors associated with smear positive tuberculosis in homeless individuals

In multivariable logistic regression, smoking cigarette regularly for greater than 5 years, BMI less than 18.5 and HIV infection were statistically significant association with smear positive PTB. Participants who smoke cigarette regularly for greater than 5 years were 10.1 times more likely to have smear positive PTB than those who smoke cigarette regularly for less than 5 years. The study also showed that statistically significant association between smear positive PTB and BMI. Participants who had BMI less than 18.5 were 6.9 times more likely to have smear positive PTB as compared to those who had BMI greater than 18.5. Furthermore, HIV infected homeless individuals were 6.8 times more likely to have smear positive PTB than those HIV uninfected homeless individuals (Table 4).

**Table 4:** Factors associated with smear positive pulmonary tuberculosis in homeless individuals Dessie and Debre Birhan towns, Northeast Ethiopia, September 2014 to June 2015 (N = 351)

Variables	Smear positive PTB		Crude odds ratio (95 % CI)	Adjusted odds ratio (95 % CI)	P value
	Negative	Positive			
Smoking					
>5 years	60	7	11.8 (1.4–98.1)	10.1 (1.1–97.7)	0.046
≤5 years	101	1	1	1	
BMI (kg/m^2^)					
<18.5	20	5	20.1 (5.0–80.8)	6.9 (1.2–41.1)	0.033
≥18.5	322	4	1	1	
HIV status					
Positive	17	5	23.9 (5.9–97.1)	6.8 (1.1–40.1)	0.036
Negative	325	4	1	1	

## Discussion

This study showed that the prevalence of smear positive PTB among TB suspect homeless individuals was 2.6 %. This finding is in line with studies conducted in USA (3.28 %) [23], Japan (1.5 %) [24] and Rome (3.86 %) [25]. This is also supported by systematic review and meta-analysis of prevalence of active TB among homeless individuals, estimated to be 0.2–7.7 % [8]. In this study all TB cases are found in young and productive age group (17–44 years) of the participants. This finding is consistent with reports from similar socio-economical settings, highest among young adults, which lead to grave socio-economic consequences in a country [26]. This high TB burden may also attribute to an aggressive transmission of TB in the homeless individuals and to the surrounding community.

Moreover, TB prevalence in this study is to some extent higher than studies conducted among homeless individuals in Marseilles (1 %) [27] and Iran (1.2 %) [15], the lower prevalence of TB in those countries could be due to high socio-economic status and low overall TB prevalence in the countries. In addition, relatively lower prevalence of TB in Iran might be due to lower prevalence of HIV infection (3.4 %) [15]. However, TB prevalence in this study is lower than studies conducted among homeless individuals in USA (6.1 %) [28], Seoul, South Korea (24.86 %) [13], North-eastern Poland (4.13 %) [29] and Colombia (7.9 %) [30], the difference might be due to difference in study design and setting, sample size and laboratory diagnosis method used. Particularly a study in USA, the analysis covered a wide geographic area with large sample size and the cases were either smear positive PTB or culture confirmed other form of TB [28]. On the other hand, in South Korea the prevalence of active TB was not based on sputum smear AFB but, it is based on chest radiography [13] this could be over sensitive and not specific enough as FM thus, might be increase the prevalence of TB than this study. In North-eastern Poland, the participants were first screened by chest radiography, then molecular testing and culture were performed [29], the use of an advanced diagnostic technique might be not underestimated the actual prevalence. In Colombia all reported cases were culture positive for MTB [30], could be revealed all types of TB in addition to smear positive PTB.

Several studies indicated that higher prevalence of TB in homeless individuals than the general population [24, 31, 32]. For example, in USA compared to the general population, homeless individuals had an approximately tenfold increase in TB prevalence [33]. Extrapolation of this study finding also indicated that about 4.67 times higher burdens of smear positive PTB in homeless individuals than the general population (108/100,000) in Ethiopia [34]. This study point prevalence of smear positive PTB is comparable to a study conducted in Greater London (780/100,000) [32]. Furthermore, despite the overall decline in TB incidence in the general population, 28 outbreaks of TB occurred in Illinois among homeless individuals, indicating that ongoing transmission of TB to the homeless individuals and the entire community [35]. This disproportionate burden of TB in homeless individuals might be due to that these groups are the most neglected and live in under-privileged social conditions such as poverty, malnutrition and overcrowd unhygienic environment with relatively limited access to health care [10].

In this study RIF resistant or MDR-TB was not found. However, studies conducted in USA (2.7 %) [28], USA (1.1 %) [33], Busan Medical Center, Korea (11.5 %) [10] and London (6.5 %) [32] MDR–TB was found among homeless individuals. Absence of MDR-TB in this study might be due to the small number of smear positive PTB cases enrolled in the study. However well known risk factors for the development of MDR-TB, previous anti-TB treatment defaulter rate is high (60 %) in this study.

In this study, participants who smoke cigarette regularly for greater than 5 years were 10.1 times more likely to have smear positive PTB than those who smoke cigarette regularly for less than 5 years. Even though there were no other studies considered the duration of smoking in homeless individuals, the role of smoking in the development of active TB is well established [36] either through increased susceptibility to new infection with MTB or increase the risk of developing active TB [37]. Thus, increasing duration of smoking might be increase the development of TB disease. Smoking cigarette by itself has also reported as a risk factor for acquisition of active TB in Rome [25] and Montreal, Canada [38] but in this study it was not significantly associated. However, in this study about 88.9 % smear positive PTB cases were found among cigarette smokers.

In addition, this study showed that participants who had BMI less than 18.5 were 6.94 times more likely to have smear positive PTB as compared to those who had BMI greater than 18.5. This finding also supported by studies conducted in Seoul, South Korea [13] and Rome [25]. In fact, malnutrition is adversely affecting the immune status of individuals; it makes individuals more susceptible to TB infection and progression of active TB disease [39]. Moreover, in this study, HIV infected homeless individuals were 6.75 times more likely to have smear positive PTB than those HIV uninfected homeless individuals. This is in line with studies conducted in USA [23, 28] and Montreal, Canada [38]. It has well known that HIV infection often leads to a greater rate of TB either through reactivation or increased susceptibility to new infection with MTB thus, the main driving factor which aggravates TB. The lifetime risk of HIV infected individuals to develop TB is 20-37 times higher than HIV uninfected individuals [17]. In addition, in this study HIV infection was found in 22 (6.3 %) of the study participants. This high HIV burden might be due to risky behaviors of homeless individuals to acquire the disease. In this study TB–HIV co-infection were also considerably high (55.56 %).

In contrast to studies conducted in USA [28], Rome [25] and Montreal, Canada [38], in this study alcohol drinking was not significantly associated with smear positive PTB. The reason might be more or less homeless individuals in this study, exhibit similar alcohol drinking characteristics (about three quarters of the participants were drunk alcohol during the study periods), this might reduce the individual variation and make it difficult to see its effect on outcomes of smear positive PTB. However, about 88.9 % smear positive PTB cases were found among alcohol drinker study participants. In addition, in contrast to a study conducted in Seoul, South Korea [13], in this study past history of TB disease is not significantly associated with smear positive PTB. The reason might be participants who had a past history of TB disease were small in number in this study, might be not enough to show the effect of past history of TB on smear positive PTB.

## Limitation of study

Smear negative PTB didn’t look for, as well as extra pulmonary TB. Probably the 2 weeks cough can be mis-determined by persons living on streets with limited date and time remembrance and others.

## Conclusion

The prevalence of smear positive PTB among TB suspect homeless individuals was high. Extrapolation of this study finding also indicates that the point prevalence of smear positive PTB in homeless individuals was 4.67 times higher than the general population in Ethiopia. It indicates that there is high transmission of TB in the homeless individuals and also become a risk to the entire community. Although MDR-TB was not found, well known risk factors for MDR-TB, previous anti-TB treatment defaulter rate is higher in the study participants. Smoking cigarette regularly for greater than 5 years, malnutrition and HIV infection were significantly associated factors with smear positive PTB among homeless individuals. Special emphasis is needed for homeless individuals to exert intensive effort to identify undetected TB cases to limit circulation of the disease into the community. Developing and implementing specific TB prevention and control strategies with integrated risk reduction approach is needed for homeless individuals.

## Abbreviations

AFB: acid fast bacilli
BMI: body mass index
CDC: Center for Disease Control and prevention
FM: fluorescence microscopy
HIV: human immunodeficiency virus
INH: isoniazid
MDR-TB: multidrug resistant tuberculosis
MTB: *M. tuberculosis*
PCR: polymerase chain reaction
PTB: pulmonary tuberculosis
RIF: rifampicin
TB: tuberculosis
